# The Analgesic Effects of Nrf2 Activators in Chemotherapy-Induced Neuropathic Pain: Evidence from Animal Studies and Consequences for Translation into Clinical Trials

**DOI:** 10.3390/ijms27041748

**Published:** 2026-02-11

**Authors:** Jimin Kim, Jeongmin Kim, Hee Kee Kim, Salahadin Abdi

**Affiliations:** 1 University of the Incarnate Word School of Osteopathic Medicine, San Antonio, TX 78235, USA; jikim13@student.uiwtx.edu; 2 Department of Epidemiology Concentration in Pharmacoepidemiology, Johns Hopkins Bloomberg School of Public Health, Baltimore, MD 21205, USA; jeongminhanakim@gmail.com; 3 Department of Science, Southern Reformed College and Seminary, Houston, TX 77084, USA; 4 Department of Pain Medicine, Division of Anesthesiology, Critical Care & Pain Medicine, The University of Texas MD Anderson Cancer Center, Houston, TX 77030, USA

**Keywords:** chemotherapy-induced neuropathic pain, Nrf2, analgesic effects, review

## Abstract

Chemotherapy-induced neuropathic pain (CINP) can be caused by several chemotherapeutic drugs, including paclitaxel, oxaliplatin, and vincristine, which is difficult to treat with several drugs, including antidepressants and anticonvulsants. The patho-mechanisms of CINP are not completely understood. However, they showed oxidative stress, mitochondrial damage, ion channel damage, and immunological dysfunction. Acting as a key regulator of antioxidant responses, nuclear factor erythroid 2-related factor 2 (Nrf2) decreased oxidative stress and mitochondrial damage. In addition, it plays a role in inhibiting nuclear factor kappa B (NF-κB). A systematic, English-only search of MEDLINE (PubMed) was performed for studies on Nrf2, chemotherapy, and neuropathic pain from database inception through 1 December 2024. Several Nrf2 activators, including tempol, oltipraz, rosiglitazone, pristimerin, cannabidiol, daidzein, bardoxolone methyl, curcumin, resveratrol, and mitoquinone, demonstrated analgesic effects in CINP animal models. Furthermore, in clinical studies, curcumin demonstrated significant efficacy in reducing vincristine-induced neuropathy in pediatric leukemia patients, while the combined administration of alpha-lipoic acid with ipidacrin hydrochloride prevented paclitaxel-induced motor neuropathy and improved axonal function in breast cancer patients. Thus, the purposes of our review article were to summarize the analgesic effects of Nrf2 activators and the patho-mechanisms of Nrf2 in CINP animal, and then the consequences for clinical trials were presented.

## 1. Introduction

Peripheral neuropathy is caused by damage to the nerves that constitute the peripheral nervous system and results in a condition with pain, stabbing, tingling, burning, weakness, and numbness in the peripheral tissues, especially the hands and feet. It can be triggered by cancer, traumatic injuries, infections, metabolic diseases (e.g., diabetes), stroke, herpes zoster, and neurotoxic drugs (e.g., chemotherapeutic drugs) [1]. Neuropathic pain arises from lesions or dysfunctions within the somatosensory nervous system with pain, including allodynia, hyperalgesia, or numbness [2]. It is treated with antidepressants, anticonvulsants, topical analgesics, and opioids, which results in a modest analgesic effect [1].

Chemotherapy-induced neuropathic pain (CINP) can be caused by several chemotherapeutic drugs, including (1) platinum drugs (cisplatin and oxaliplatin), (2) vinca alkaloids (vincristine, vinblastine, and vinorelbine), (3) anti-microtubule agents (taxanes, including paclitaxel, and docetaxel), (4) proteasome inhibitors (bortezomib), and (5) immunomodulatory agents (thalidomide and lenalidomide) [3]. CINP stands as a major, enduring complication of chemotherapy, impacting an estimated 30–40% of patients annually [4]. This serious adverse effect can lead to premature cessation of chemotherapy or dose reductions, ultimately compromising treatment efficacy and patient survival rates [5]. The intensity of CINP is influenced by the duration of chemotherapeutic drugs [6]. Despite its significant clinical impact, the treatment of CINP represents a considerable medical challenge, primarily stemming from the absence of agents recommended for its prevention and management within the American Society for Clinical Oncology clinical practice guidelines [7]. The patho-mechanisms of CINP are not completely understood; however, oxidative stress has been reported as one of its patho-mechanisms, which means nuclear factor erythroid 2-related factor 2 (Nrf2) activators show analgesic effects in CINP. The previous two review articles were reported on Nrf2 in preclinical models of chronic pain [8,9]. Therefore, the purposes of our review article were to summarize the analgesic effects of Nrf2 activators on preclinical and clinical studies with CINP and patho-mechanisms of Nrf2.

### 1.1. Patho-Mechanisms of CINP

**Figure 1:**
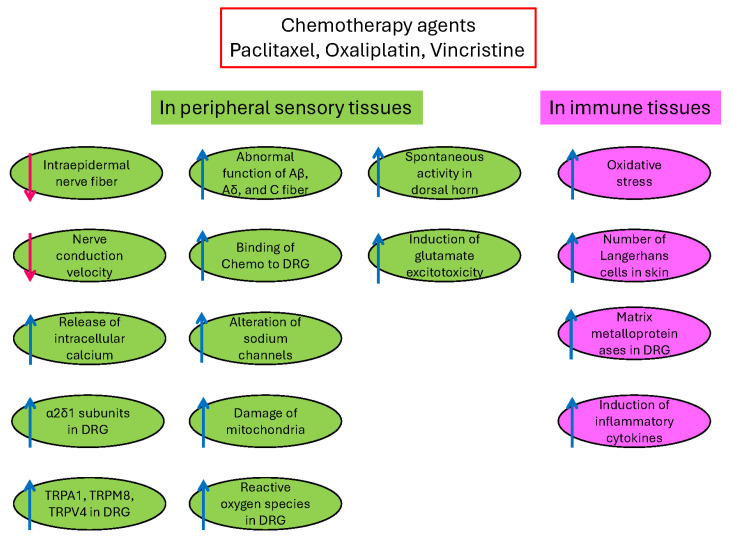
The mechanisms of chemotherapy-induced neuropathic pain (CINP). Chemotherapy agents induce changes in peripheral sensory tissues (**left**) and immune tissues (**right**). ↑, increase; ↓, decrease; green, sensory tissues; pink, immune tissues.

Platinum drugs and proteasome inhibitors can damage sensory nerves [10]. In addition, others, including vinca alkaloids and anti-microtubule agents, can damage both sensory and motor nerves [10]. They destroyed peripheral nervous tissues like dorsal root ganglia (DRG), skin nerve terminals, and primary sensory neurons [10]. The mechanisms of CINP include: (1) decreases of the number of intraepidermal nerve fibers in skin nerve terminals; (2) increases of abnormal function of Aβ, Aδ, and C fibers by vincristine; (3) the slowing nerve conduction velocity by cisplatin, paclitaxel, and bortezomib; (4) binding of paclitaxel and oxaliplatin to the DRG; (5) alteration of the function of voltage-gated sodium channels by oxaliplatin; (6) damage of mitochondria by paclitaxel, vincristine, cisplatin, and bortezomib; (7) release of intracellular calcium; (8) increases of α2δ1 subunits of voltage-gated calcium channels in the dorsal horn and DRG; (9) upregulation of transient receptor potential (TRP) vanilloid 1, TRPA1, TRPM8, and TRPV4 in the DRG by cisplatin, oxaliplatin, and paclitaxel; (10) increases of reactive oxygen species in the DRG by cisplatin, oxaliplatin, and paclitaxel; (11) increases of spontaneous activity and after-discharges in the dorsal horn by paclitaxel; and (12) induction of glutamate excitotoxicity [3] (Figure 1).

In addition, chemotherapeutic agents can affect immune cells, including macrophages, epidermal Langerhans cells, and glial cells. They produce (1) increases in oxidative stress, (2) increases in the number of Langerhans cells in the skin, (3) induction of matrix metalloproteinases in DRG, (4) activation of macrophages in DRG, and (5) induction of inflammatory cytokines [3]. Oxidative stress is one of the underlying patho-mechanisms of CINP [9]. Furthermore, Nrf2 (nuclear factor erythroid 2-related factor 2) is a critical regulator of oxidative stress and decreases oxidative stress [11] (Figure 1). Thus, the Nrf2 activator contributed to the analgesic effects of CINP [11].

### 1.2. Action of Nrf2 Activators in CINP

Characterized by an imbalance, oxidative stress involves an excess of free radicals—reactive oxygen species (e.g., superoxide anions and hydrogen peroxide) and reactive nitrogen species (nitric oxide)—relative to the available low levels of antioxidants, including superoxide dismutase (SOD), nitric oxide synthase, glutathione peroxidase (GPX), vitamin C, and vitamin E [12].

In CINP, chemotherapeutic agents, including paclitaxel, induced free radical production and mitochondrial dysfunction in the peripheral nerve [13]. Free radicals are produced by enzymes in nervous cells and immune cells, including macrophage, neutrophil, lymphocyte, and dendritic cells [14]. In addition, oxidative stress produced proinflammatory inducers, which include pro-inflammatory cytokines and adhesion molecules, by the activation of nuclear factor kappa B (NF-κB) [15]. By initiating the degradation of IκB, a protein that normally inhibits NF-κB, leading to the translocation of NF-κB (p50 and p65 subunit) to the nucleus and then activation of proinflammatory responses, oxidative stress can lead to the activation of NF-κB, which induces PINP [11].

**Figure 2:**
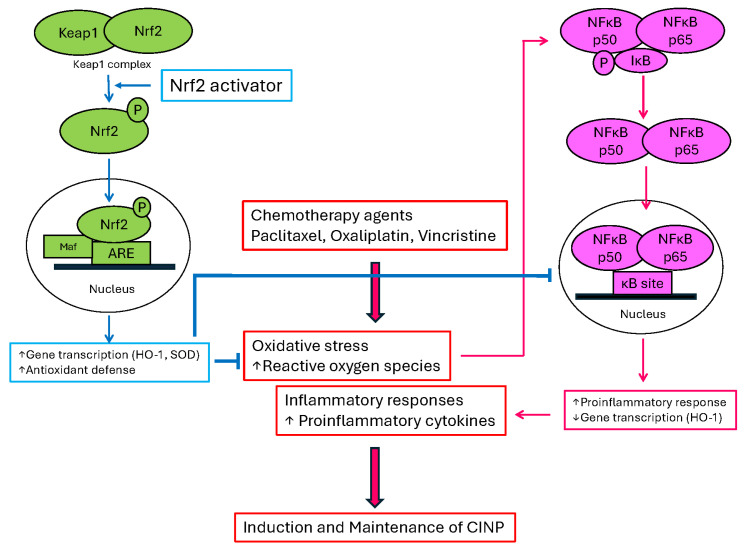
Cartoon model of nuclear factor erythroid 2 (NFE2)-related factor 2 (Nrf2) signaling and nuclear factor kappa B (NF-κB) in chemotherapy-induced neuropathic pain (CINP). Chemotherapy agents induce neuropathic pain by increasing both oxidative stress and inflammatory responses (center). Nrf2 activator decreases oxidative stress by initiating several cytoprotective genes (SOD and HO-1) and proinflammatory responses by inhibiting the NF-κB (p50 and p65 subunit) pathway, which can ameliorate CINP and prevent the induction of CINP. ⬇, induce; ↓, decrease; ↑, increase; ⊣, block.

Nrf2 is produced by the NFE2L2 gene. Nrf2 contains a bZip domain at the C-terminus that is responsible for the formation of heterodimers with other bZip proteins, such as small muscle aponeurosis fibromatosis (MAF) [16]. These heterodimers are the regulators of human genes, including the antioxidant response element (ARE), which resembles the NFE2-binding motif [16]. In neuropathic pain conditions, Nrf2 is released from Keap1 and translocates to the nucleus, where it heterodimerizes with MAF proteins. The complex Nrf2–MAF binds to ARE, initiating the transcriptions of several cytoprotective genes, such as heme oxygenase-1 (HO-1), NAD(P)H:quinone oxidoreductase1 (NQO1), SOD, glutathione cysteine ligase, glutathione S-transferases, and catalase, which induce antioxidant defense [17]. Nrf2 activators decreased oxidative stress by inhibiting the translocation of NF-κB to the nucleus, which produces analgesic effects in CINP (Figure 2). We conducted this review as a State-of-the Art Narrow Reviews.

## 2. Results and Discussion

### 2.1. Analgesic Effects of Nrf2 Activators in CINP: Preclinical (Animal) Studies

#### 2.1.1. Paclitaxel-Induced Neuropathic Pain (PINP) Model

The collection of studies explored various therapeutic approaches to alleviate CINP, a common and debilitating side effect of chemotherapy drugs like paclitaxel. Several studies indicate that activating Nrf2 plays a significant role in mitigating PINP (Table 1).

In the PINP rat, electroacupuncture at Neiguan and Jianshi acupoints reduced mechanical and thermal hypersensitivity by modulating Nrf2-ARE and increasing SOD in the DRG [18]. Similarly, combined administration of tempol (SOD mimetic), vitamin C, and GKT137831 (NADPH oxidase inhibitor) also restored Nrf2-ARE signaling pathway and then attenuated mechanical and thermal hypersensitivity [18].

Oltipraz, an Nrf2 activator, reduced mechanical allodynia and significantly alleviated PINP after repeated oltipraz injections by increasing Nrf2 in the spinal cord [19]. Furthermore, early oltipraz treatment delayed the onset of PINP, but it did not prevent the pain from developing [19].

Rosiglitazone, a peroxisome proliferator-activated receptor γ (PPARγ) agonist, alleviated established PINP and delayed the onset of neuropathic pain by increasing Nrf2 expression [20]. Furthermore, these analgesic effects were reversed by both PPARγ antagonist and Nrf2 inhibitor, confirming the roles of PPARγ and Nrf2 in this process [20].

Hydrogen-rich water (HRW) was administered systemically to reduce mechanical and thermal allodynia in PINP and improve memory and alleviate anxiety- and depressive-like behaviors by modulating the Nrf2 pathway [21].

Pristimerin, one of the triterpenes, showed promise in promoting Nrf2 activation, a key antioxidant defense, and in inhibiting monoacylglycerol lipase activity, preventing the development of PINP in mice [22]. Similarly, the combination of cannabidiol (CBD) and tetrahydrocannabivarin (THCV) demonstrated neuroprotective effects by modulating Nrf2 in DRG and improving behavioral outcomes [23].

Daidzein (DZ), a soy isoflavone, activated the Nrf2 pathway, leading to enhanced antioxidant enzyme expression and diminished neuronal apoptosis in CINP models [24]. It also alleviated pain hypersensitivity, reduced TRPV1 and P2Y receptor expression, and activated Nrf2-related antioxidant pathways, while also decreasing neuroinflammation and apoptosis [24]. The Commiphora myrrha (CM) extract provided protection against paclitaxel-induced hyperalgesia and allodynia, showing similar benefits via peripheral TRPV1 inhibition and the restoration of Nrf2 [24].

Other studies demonstrated that bardoxolone methyl (BM), an Nrf2 activator, provided analgesic effects by restoring Nrf2 expression, reducing inflammation, and improving mitochondrial function in DRG neurons [9]. In a related study, resolvin D1 (RvD1), a lipid mediator, alleviated PINP by regulating immune responses through N-formyl peptide receptor 2 and IL-10, activating Nrf2-HO1 signaling pathways in DRG neurons, and reducing oxidative stress and neuronal damage [25].

A combination therapy of cobalt protoporphyrin IX (CoPP) with HRW demonstrated enhanced efficacy in treating PINP, improving both pain and emotional disorders by reducing inflammation and activating the HO-1/Nrf2 axis [26].

Finally, caffeic acid phenethyl ester (CAPE), a phenolic compound, alleviated PINP by inhibiting β-catenin, an important Wnt signaling protein; reducing the expression of inflammatory markers such as matrix metalloproteinase 2; and promoting Nrf2 activation [27].

They underscore that activating Nrf2 could be a therapeutic strategy to prevent and alleviate PINP by modulating oxidative stress, inflammation, and cellular damage. Thus, Nrf2 represents a promising target for future therapies aimed at treating PINP.

**Table 1:** Analgesic effects of Nrf2 activators in paclitaxel-induced neuropathic pain (PINP).

Treatment	Mechanisms of Action	Analgesic Effects	Reference
Electroacupuncture (EA)	EA modulates Nrf2-antioxidant response element (Nrf2-ARE) and increases the expression of superoxide dismutases (SOD) in the dorsal root ganglion (DRG).	EA significantly reduced mechanical and thermal hypersensitivity in PINP rats	[18]
Tempol (SOD mimetic, 20 mg/kg, ip), vitamin C (500 mg/kg), GKT137831 (NADPH oxidase inhibitor, 1 mg/kg)	The treatments restore Nrf2-ARE signaling, increase SOD levels, and reduce proinflammatory cytokines (IL-1β, IL-6, and TNF-α) in the DRG.	The treatments significantly attenuated mechanical and thermal hypersensitivity in PINP rats.	[28]
Oltipraz (Nrf2 activator, 10, 50, 100 mg/kg during days 14–18, ip)	Oltipraz activates Nrf2 and upregulates Heme oxygenase 1 (HO-1) in the spinal cord.	Oltipraz reduced mechanical allodynia in PINP rats.	[19]
Rosiglitazone (PPARγ agonist, 50 mg/kg daily ip during days 14–18)	Rosiglitazone activates peroxisome proliferator-activated receptor γ (PPARγ) and activates the Nrf2/HO-1 pathway in the spinal cord.	Rosiglitazone alleviated established PINP and delayed the onset of neuropathic pain.	[20]
Hydrogen-rich water (HRW)	HRW modulates Kv7 potassium channels and the Nrf2-HO-1-NAD(P)H: quinone oxidoreductase 1 pathway.	HRW reduced mechanical and thermal allodynia in PINP.	[21]
Pristimerin (0.25, 0.5, 0.75, 1 mg/kg, ip, days 1–4, 7–11)	Pristimerin upregulates Nrf2 and inhibits monoacylglycerol lipase activity.	Pristimerin prevented mechanical allodynia in PINP.	[22]
Cannabidiol (CBD, 10 mg/kg, ip, twice a week for 6 weeks) and Tetrahydrocannabivarin (THCV, 15 mg/kg, ip, twice a week for 6 weeks)	Combination therapy of CBD and THCV modulates Nrf2 in DRG and improves mitochondrial function by reducing superoxide levels.	The combination of CBD and THCV improved both thermal and mechanical hypersensitivity in PINP.	[23]
Daidzein (DZ, 0.1, 1, 10 mg/kg, ip, days 8–14)	DZ (1) downregulates TRPV1 channels and P2Y purinergic receptors, (2) activates the Nrf2/HO-1, (3) reduces neuronal apoptosis, and (4) reduces the production of pro-inflammatory mediators.	DZ significantly alleviates pain hypersensitivity in PINP, improving mechanical and thermal thresholds in behavior tests. In addition, DZ reverses histological damage caused by paclitaxel and inhibits the increase in vascular permeability.	[24]
Commiphora myrrha (CM) resin extract (250 mg/kg, oral, days −7~−3, 0–4)	CM inhibits the TRPV1 activity in the spinal cord and increases the expression of Nrf2 in the paw skin of mice.	CM reduced thermal hyperalgesia and mechanical allodynia and prevented the development of PINP.	[24]
Resolvin D1 (RvD1, 5 μg/kg, ip for days 0–16)	RvD1 activates N-formyl peptide receptor 2, increases IL-10 production in macrophages, and activates the Nrf2-HO-1 in DRG.	RvD1 reduced mechanical pain hypersensitivity in PINP mice.	[25]
Bardoxolone methyl (BM, 10 mg/kg, ip, days 21–24)	BM activates Nrf2, increases phosphorylated Nrf2 (pNrf2), reduces inflammatory mediators, and restores mitochondrial function.	BM treatment effectively ameliorates PINP in rats, both after a single injection and with repeated injections	[11]
The combined treatment of CoPP (a HO-1 inducer) and hydrogen-rich water (HRW)	CoPP promotes HO-1 expression and Nrf2 activation. HRW reduces oxidative stress. The combination decreases the activation of the NLRP3 inflammasome and reduces oxidative markers such as 4-hydroxynonenal in the DRG and amygdala. In addition, the combination treatment increases expression of Nrf2, HO-1, SOD 1, and glutathione S-transferase mu 1 in the DRG.	The combination treatment of CoPP and HRW significantly reduced mechanical and thermal allodynia compared to either treatment alone, showing faster and stronger effects in alleviating pain. In addition, the combined treatment also reduced anxiodepressive-like behaviors associated with CINP.	[26]
Caffeic acid phenethyl ester (CAPE, 10, 30 mg/kg, ip, days 21–28)	CAPE reduces β-catenin, a key component of the Wnt signaling pathway and decreases matrix metalloproteinases-2, which is associated with tissue remodeling and inflammation in neuropathic pain. It also increases the expression of Nrf2 against oxidative stress.	CAPE improved the pain threshold in PINP rats.	[27]

#### 2.1.2. Oxaliplatin-Induced Neuropathic Pain (OINP) Model

Oxaliplatin, a widely used chemotherapeutic agent, frequently induces peripheral neuropathy characterized by pain and sensory dysfunction, largely driven by oxidative stress and inflammation. Through several studies, various strategies targeting the antioxidant pathway—particularly involving Nrf2—demonstrated protective or therapeutic effects against OINP. Nrf2 plays a pivotal role in mitigating this neurotoxicity, as highlighted by multiple studies (Table 2).

Inhibition of microRNA-155 restored Nrf2-ARE signaling and reduced oxidative markers and pain behaviors [29]. Natural compounds like puerarin, resveratrol, and curcumin enhanced Nrf2 activity and its downstream targets such as glutathione peroxidase 4 (GPX4), HO-1, and NQO1, mitigating oxidative stress and inflammation while improving pain thresholds and motor coordination [29,30,31]. Puerarin and curcumin enhanced Nrf2/GPX4 signaling, suppressed inflammatory responses via the NLRP3 inflammasome, and alleviated pain symptoms [31,32]. Resveratrol further amplified Nrf2 activity, boosting the expression of HO-1, while attenuating neuroinflammation and behavioral hypersensitivity [30]. Additionally, bone marrow-derived mesenchymal stem cells reversed OINP by upregulating Nrf2 and antioxidant enzymes like SOD, along with anti-inflammatory cytokines IL-10 and TGF-β [33].

Collectively, the results emphasize Nrf2’s critical role in regulating oxidative stress and neuroinflammation in OINP and support antioxidant-focused therapies as promising approaches for managing chemotherapy-induced peripheral neuropathic pain.

**Table 2:** Analgesic effects of Nrf2 activators in oxaliplatin-induced neuropathic pain (OINP).

Treatment	Mechanisms of Action	Analgesic Effects	Reference
miR-155 inhibitor (2 μg/day, intrathecal injection, days 0–5)	Inhibition of miR-155 restores Nrf2-ARE signaling in the dorsal horn and suppresses NOX4. This also decreased TRPA1 upregulation.	The treatment significantly attenuated mechanical allodynia and cold hyperalgesia in OINP.	[29]
Puerarin (Pue, 10 mg/kg, ip, days 15–21)	Pue activates Nrf2, increases its association with glutathione peroxidase 4 (GPX4), and increases antioxidative elements within the spinal cord. Additionally, this suppresses the NLRP3 inflammasome-mediated inflammatory responses.	Pue improved pain hypersensitivity (mechanical pain threshold and thermal latency), spontaneous pain, and motor coordination in OINP mice.	[32]
Resveratrol (RESV, oral 7, 14 mg/kg/day during days 4–17)	RESV, a natural antioxidant, works by modulating key antioxidant and anti-inflammatory pathways, including upregulating Nrf2, HO-1, restoration of the GSH/GSSG ratio, reduction of the expression of NFκB and TNFα, and reduction of neuronal injury markers like ATF3 and c-fos in the spinal cord and dorsal root ganglia.	RESV administration prevented mechanical and thermal allodynia in OINP.	[30]
Curcumin (CUR, 100, 200 mg/kg, oral)	CUR reduces NLRP3-mediated inflammation in the spinal cord.	Cur alleviated ONIP.	[31]
Mesenchymal stem cells (MSC, single iv injection 10^6^ cells on day 6)	MSCs increase the levels of anti-inflammatory cytokines like IL-10 and TGF-β in the spinal cord, enhance SOD and Nrf-2, and decrease nitrite and malondialdehyde levels.	MSC treatment completely reversed mechanical allodynia and thermal hyperalgesia in OINP. In comparison, gabapentin provided only transient relief.	[33]

#### 2.1.3. Vincristine-Induced Neuropathic Pain (VINP) Model

Vincristine, a common chemotherapeutic agent, induces neuropathic pain through mechanisms such as oxidative stress and inflammation. In studies investigating therapeutic options, compounds like levo-corydalmine (l-CDL), mitoquinone, and ajugarin-I show potential for alleviating VINP, as shown in Table 3 [34,35,36]. l-CDL activates the Nrf2, thereby reducing neuroinflammation and preventing nerve damage [34]. Mitoquinone, a mitochondrial-targeted antioxidant, improves VINP by reducing oxidative stress, restoring mitochondrial function, and protecting against neuronal apoptosis [35]. Ajugarin-I, derived from Ajuga bracteosa, mitigates neuropathy through its antioxidant and anti-inflammatory properties by regulating Nrf2/NF-κB and Bcl-2 signaling pathways [36]. These findings highlight Nrf2 activation as a common therapeutic target for preventing vincristine-induced neuropathy, reducing oxidative stress, and enhancing neuroprotection. This activation of Nrf2 helps in preventing neuronal damage, improving mitochondrial function, and alleviating pain, making it a potential therapeutic target for VINP.

### 2.2. Clinically Available Nrf2 Activators

The identification of effective Nrf2 activators is a major research focus for numerous diseases, including CINP. These compounds can be broadly classified into natural compounds (phytochemicals) and synthetic compounds, many of which have been studied in preclinical CINP models, with some progressing to clinical trials for other indications. In clinical studies in Table 4, curcumin showed significant efficacy in reducing vincristine-induced neuropathy in pediatric lymphoblastic leukemia patients, while the combination of alpha-lipoic acid and ipidacrin hydrochloride improved axonal function, preventing motor neuropathy induced by paclitaxel in breast cancer patients [37,38]. Both compounds, possessing Nrf2 activation, represent potential protective agents against neurotoxicity caused by different chemotherapy drugs.

In detail, the authors indicated that the prevalence of vincristine-induced peripheral neuropathy was substantially lower in the curcumin-treated group compared to the placebo group, as confirmed by both nerve conduction studies and needle electromyography assessments. Furthermore, curcumin demonstrated a protective effect against motor and sensory nerve damage, with a significant reduction in motor nerve abnormalities [35]. Natural curcumin and curcuminoids like monocarbonyl analogues produce antitumor actions acting on the following nuclear targets, including NF-κB, STAT3, topoisomerase IIα, and PARP. In addition, they influenced HIF-1α and then blocked angiogenesis. Furthermore, they disrupted tumor cell energy metabolism by inhibiting key glycolytic enzymes and reducing anaerobic energy production [39].

In breast cancer patients (stage II–IV) treated with paclitaxel with doxorubicin or epirubicin, alpha-lipoic acid, and ipidacrin hydrochloride tendency toward axonal damage and mild myelinopathy and significantly increased M-response rates of motor nerves after 6 cycles of polychemotherapy with paclitaxel [36].

Given its strong antioxidant, curcumin and alpha-lipoic acid may serve as a promising adjunct therapy for mitigating CINP.

#### 2.2.1. Natural Nrf2 Activators (Phytochemicals)

These compounds are derived from plants and are often found in diets. They are generally perceived to have lower toxicity owing to their natural origin and dietary presence. They typically activate Nrf2 by interacting with specific cysteine residues on Keap1, leading to Nrf2 release and nuclear translocation. However, high doses, specific formulations or interactions with concurrent medications can still lead to adverse effects. In Table 4, natural Nrf2 activators were shown, including sulforaphane, curcumin, resveratrol, quercetin, alpha-lipoic acid, oltipraz, and berberine (Table 4).

#### 2.2.2. Synthetic Nrf2 Activators

These compounds are designed and synthesized to specifically target and activate the Nrf2 pathway, often by disrupting the Keap1-Nrf2 interaction. These are generally more potent and selective, necessitating rigorous safety evaluation given their pharmaceutical nature. In Table 4, synthetic Nrf2 activators were shown, including dimethyl fumarate (DMF), monomethyl fumarate, omaveloxolone, and bardoxolone methyl (BM). Among them, DMF and omaveloxolone have approval from the FDA for multiple sclerosis and Friedreich’s ataxia, respectively, which means they have a well-characterized safety profile for chronic neurological conditions [40,41]. Therefore, they may be highly attractive candidates for CINP by conducting small pilot studies or an off-label study.

**Table 4:** Nrf2 activators for clinical study with CINP or other diseases.

Name	Mechanisms	Safety	Reference
Curcumin (N)	Direct Keap1 modification and modulation of upstream signaling	Generally Well-Tolerated	[42]
	Clinical trial Disease: Acute Lymphoblastic Leukemia Condition: Vincristine 1.5 mg/m^2^ weekly Patient: Pediatric male and female, 5–15 years old Curcumin: 3 mg/kg twice daily, oral for 3 months Result: The results showed that curcumin is effective in preventing the development of vincristine-induced peripheral neuropathy and leads to its improvement in these patients. Adverse events: mild gastrointestinal symptoms (diarrhea, anorexia, constipation, and vomiting). No significant difference was observed in terms of gastrointestinal complications between the curcumin and placebo groups.		[37]
Alpha-Lipoic Acid (N)	Potent antioxidant and indirect activator of Nrf2	Generally Well-Tolerated	[43]
	Clinical trial Disease: Breast cancer (stage II and III) Condition: Doxorubicin 60 mg/m^2^ + cyclophosphamide 600 mg/m^2^ + paclitaxel 80 mg/m^2^ Patient: Female, 36–63 years old Alpha-lipoic acid: 600 mg/day, oral for 6 months with ipidacrine hydrochloride Results: Alpha-lipoic acid may represent a promising adjuvant therapy to attenuate paclitaxel-associated neuropathy and doxorubicin-induced cardiotoxicity in women with breast cancer. Adverse events: headache, nausea, abdominal discomfort, and abdominal pain. There were similar adverse events in the chemotherapy regimen.		[38]
Sulforaphane (N)	Potent electrophilic Nrf2 activator via Keap1 modification	Generally Regarded as Safe	[44]
	Clinical trial Disease: Lung cancer Condition: Former smokers (high risk) Patient: male and female, 55–75 years old Sulforaphane: 95 μmol/day, oral for 12 months Results: This study demonstrated that oral supplementation of sulforaphane for 12 months significantly reduced the Ki-67 index, a potential surrogate endpoint of biomarkers for lung cancer risk. Adverse events: 85% of the sulforaphane group and 91% of the placebo group reported gastrointestinal adverse events, including flatulence, followed by constipation, diarrhea, and dry mouth. There were 71% of the sulforaphane group and 68% of the placebo group reported neurological adverse events such as dizziness, headache, and mood change.		[45]
Resveratrol (N)	direct Nrf2 activation and involvement of SIRT1	Generally Well-Tolerated	[46]
	Clinical trial Disease: Polycystic ovary syndrome Condition: Assisted reproduction Patient: Female, 18–35 years old Resveratrol: 800 mg/day, oral for 2 months Results: This study indicated that resveratrol may be a promising therapeutic agent for patients with polycystic ovary syndrome undergoing assisted reproduction. Adverse events: Resveratrol was well tolerated throughout the treatment, with no patients reporting any side effects.		[47]
Quercetin (N)	Direct interaction with Keap1 and modulation of signaling pathways	Generally safe for 12 weeks	[48]
	Clinical trial Disease: Polycystic ovary syndrome Condition: Assisted reproduction Patient: Female, 18–35 years old Quercetin: 500 mg/day, oral for 40 days Results: Quercetin consumption causes improvement in oocyte and embryo grade and the pregnancy rate. Adverse events: No major side effects with quercetin were reported.		[49]
Oltipraz (N)	The electrophile that modifies Keap1 cysteine residues	Dermatological issues (skin irritation, photosensitivity), gastrointestinal disturbances, and fatigue	[50]
	Clinical trial Disease: Colorectal cancer Condition: Risk for colorectal cancer Patient: Male and female, 46–82 years old Oltipraz: 125 or 250 mg/m^2^ twice weekly for 12 weeks Results: The 125 mg/m^2^ was tolerated in patients. Adverse events: Two of seven patients at 250 mg/m^2^ produced significant fatigue.		[51]
Berberine (N)	Nrf2 activation and modulation of upstream kinases	Generally Safe at moderate doses	[52]
	Clinical trial Disease: Colorectal adenoma Condition: Risk for colorectal cancer Patient: Male and female, 60–71 years old Berberine: 0.3 g twice daily for 2 years Results: Berberine may serve as a potential long-term preventive agent against adenoma recurrence after polypectomy. Adverse events: No severe adverse events		[53]
Dimethyl Fumarate (DMF) and Monomethyl Fumarate (MMF) (S)	Electrophilic compounds that covalently modify Keap1 cysteine residues, leading to Nrf2 release	Serious Side Effects: lymphopenia and liver injury	[40]
	Clinical trial Disease: Cutaneous T-cell Lymphoma (CTCL; Stages Ib to IV) Patient: Male and female, >18 years old DMF: The dose was escalated weekly by 30 mg/day up to 120 mg/day for 24 weeks Results: This study presents DMF as an effective and excellently tolerable therapeutic option in CTCL to be further evaluated in a phase 3 study or real-life patient care, as well as in combination therapies. Adverse events: Three patients experienced an adverse event that required the drug to be withdrawn. The main side effects observed under the study were diarrhea (52.2% of the patients experienced diarrhea at least once), eosinophilia (21.7%), pain in extremity (21.7%), flushing (21.7%), upper abdominal pain (17.4%), fatigue (17.4%), pruritus (17.4%), and nasopharyngitis (17.4%).		[54]
Omaveloxolone (S)	Potent electrophilic Nrf2 activator via Keap1 modification	Serious Side Effects: Liver Injury, lipid abnormalities	[41]
	Clinical trial Disease: Friedreich Ataxia (FA) Patient: Male and female, 16–40 years old Omaveloxolone: 150 mg/day, oral for 48 weeks Results: Omaveloxolone significantly improved neurological function compared to placebo and was generally safe and well-tolerated. Adverse events: A total of 3 patients in the omaveloxolone group and 2 patients in the placebo group reported severe adverse events (SAEs) during the treatment period. Two additional patients in the omaveloxolone group experienced SAEs approximately two weeks after their final dose. Four patients receiving omaveloxolone and 2 patients receiving placebo stopped treatment due to adverse events. Notably, no serious adverse events or treatment-halting adverse events were observed in the pediatric participants during this study.		[55]
Bardoxolone Methyl (BM) (S)	Potent activator for Nrf2	Safety Issue: increased risk of heart failure.	[56]
	Clinical trial Disease: Advanced Solid Tumors and Lymphomas Patient: Male and female, >18 years old BM: 300, 600, 900 mg/day, oral for 21 days Results: Bardoxolone methyl was well-tolerated with a maximum tolerated dose of 900 mg/d. A complete tumor response occurred in a mantle cell lymphoma patient, and a partial response was observed in an anaplastic thyroid carcinoma patient. The estimated glomerular filtration rate was also increased. Adverse events: The dose-limiting toxicities were grade 3 reversible liver transaminase elevations.		[57]

Notes: N: Natural Nrf2 Activators; S: Synthetic Nrf2 Activators.

#### 2.2.3. Considerations in Nrf2 CINP Clinical Trials

There are several considerations in clinical trials, including oncological priority, the complexity of CINP, study design challenges, and safety in cancer patients. For the oncological priority, the primary focus in oncology remains cancer treatment, and interventions for side effects must not compromise anti-cancer efficacy. Nrf2 activation can sometimes confer chemoresistance to cancer cells, which is a major concern. The complexity of CINP with varied etiologies depends on chemotherapy drug. Study design is challenging due to the variability in chemotherapy regimens and outcome measures. In addition, safety in cancer patients due to their multiple comorbidities.

### 2.3. Limitations

This review is subject to several limitations. First, the limited number of studies, particularly clinical trials, prevents definitive confirmation of the efficacy of Nrf2 activators for neuropathic pain. Second, it remains unclear whether Nrf2 activators are directly involved in the underlying mechanisms of chemotherapy-induced neuropathic pain. Finally, the use of Nrf2 activators in oncology is further complicated by the lack of clarity regarding their potential anticancer effects.

### 2.4. Future Prospects

Future research efforts should focus on overcoming the current limitations to advance Nrf2 activators for clinical use. First, there is an urgent need for well-designed, dedicated clinical trials to establish the efficacy and safety of Nrf2 activators in human patients with chemotherapy-induced neuropathic pain. Second, mechanistic studies are required to clarify Nrf2’s precise role in the initiation versus the chronic maintenance of CINP pathways. Finally, investigations must prioritize clarifying the interaction between Nrf2 activators and standard chemotherapy regimens to ensure these compounds do not compromise anticancer efficacy, potentially through developing localized or tumor-specific delivery methods.

## 3. Materials and Methods

**Figure 3:**
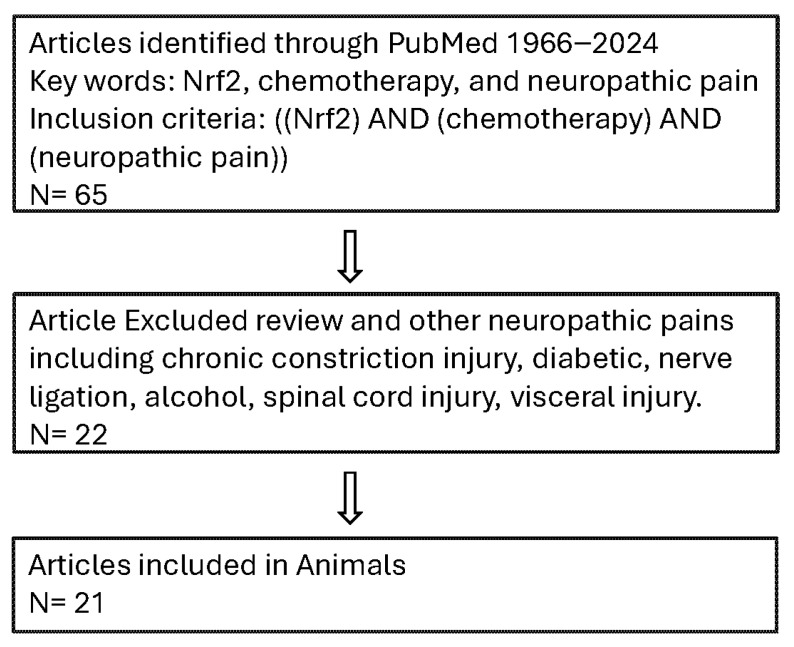
Search strategy. ↓, next step.

We conducted a reproducible, English-only systematic search of MEDLINE (PubMed) for all relevant studies published since its inception until the search execution date of 1 December 2024. The search focused on combining terms for Nrf2, chemotherapy, and neuropathic pain, with the inclusion criteria being ((Nrf2) AND (chemotherapy) AND (neuropathic pain)) from 1966 to 2024, sorted by publication date. We strictly limited the results to Animal and clinical Studies. The subsequent screening, performed by two independent authors, enforced stringent exclusion criteria against review articles and non-chemotherapy-induced neuropathic pain models, including chronic constriction injury, diabetic neuropathy, nerve ligation, alcohol, spinal cord injury, and visceral injury, to guarantee topic specificity. The search strategy is shown in Figure 3.

## 4. Conclusions

Paclitaxel, oxaliplatin, and vincristine, potent chemotherapeutic agents, lead to the development of CINP, which significantly impacts patients’ quality of life. Oxidative stress and inflammation play central roles in CINP. Targeting pathways, such as Nrf2, which regulate antioxidant responses and inflammatory responses, has shown promising therapeutic potential in attenuating these painful conditions. In CINP studies, the Nrf2 activators, including electroacupuncture, tempol, vitamin C and GKT137831, oltipraz, rosiglitazone, hydrogen-rich water, pristimerin, cannabidiol and tetrahydrocannabivarin, daidzein, Commiphora myrrha extract, resolving D1, bardoxolone methyl, caffeic acid phenethyl ester, miR-155 inhibitor, puerarin, resveratrol, curcumin, Levo-corydalmine, mitoquinone, and ajugarin-I, produced analgesic effects in animals. In clinical studies, curcumin and alpha-lipoic acid demonstrated significant efficacy in reducing CINP. Furthermore, modulation of inflammatory cytokines and apoptotic pathways may enhance neuroprotection, providing a potential avenue for creating effective therapies for CINP. Targeting the Nrf2 pathway offers a strategy for mitigating CINP, thus improving patient outcomes and quality of life during cancer treatment.

**Figure 4:**
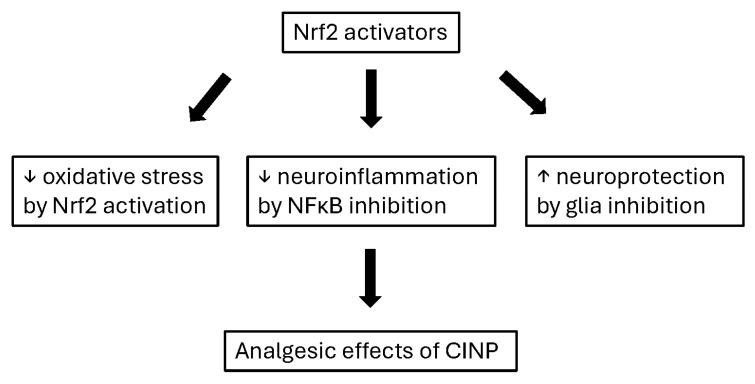
The concept of analgesic effects of NRf2 activators in CINP. ⬇, induce; ↓, decrease; ↑, increase.

Given the data, Nrf2 activators may produce analgesic effects in CINP by decreasing oxidative stress and neuroinflammation as well as increasing neuroprotection. In addition, their difficulties may be low bioavailability and the possibility of cancer cell proliferation (Figure 4). Improving the bioavailability of Nrf2 activators (e.g., curcumin, sulforaphane, and resveratrol) focuses on overcoming their poor absorption, rapid metabolism, and low water solubility. Key strategies include using nano-formulations, liposomes, and self-microemulsifying drug delivery systems to increase plasma concentrations, as seen with significant, often manifold, increases in bioavailability in studies [58,59,60].

## Figures and Tables

**Table 3:** Analgesic effects of Nrf2 activators in vincristine-induced neuropathic pain (VINP).

Treatment	Mechanisms of Action	Analgesic Effects	Reference
Levo-corydalmine (l-CDL, 5, 10, and 20 mg/kg for 9 days from the last vincristine injection)	l-CDL activates the Nrf2/HO-1/carbon monoxide pathway and improves mitochondrial function in sensory fibers (A-fibers and C-fibers) and inhibits Connexin 43-mediated pathways.	l-CDL reduced pain hypersensitivity and sciatic nerve degeneration in VINP.	[34]
Mitoquinone (MitoQ, a mitochondrial-targeted antioxidant, 2.5, 5 and 10 mg/kg once a day for days 6–14)	MitoQ enhances Nrf2 expression in the nucleus, reduces oxidative stress, decreases pro-inflammatory cytokines, inhibits mitochondrial fission (Drp1 and Fis), improves mitochondrial fusion and function, and reduces apoptosis.	MitoQ reduced pain hypersensitivity and glial activation in VINP.	[35]
Ajugarin-I (Aju-I, 1, 5 mg/kg, ip, days 11–21)	Aju-I upregulates Nrf2, suppresses NF-κB, reduces apoptosis in neuronal tissues, and restores the balance between antioxidant and oxidative stress factors in the spinal cord and sciatic nerve.	Aju-I treatment significantly alleviated hyperalgesia and allodynia in VINP mice. In addition, Aju-I reversed histological damage in the sciatic nerve, spinal cord, and brain caused by vincristine.	[36]

## Data Availability

No new data were created or analyzed in this study.

## Abbreviations

The following abbreviations are used in this manuscript:

ARE	antioxidant response element
BM	bardoxolone methyl
CAPE	caffeic acid phenethyl ester
CBD	cannabidiol
CINP	chemotherapy-induced neuropathic pain
CM	Commiphora myrrha
CoPP	cobalt protoporphyrin IX
DRG	dorsal root ganglia
DZ	Daidzein
GPX	glutathione peroxidase
GPX4	glutathione peroxidase 4
HO-1	heme oxygenase-1
HRW	Hydrogen-rich water
l-CDL	levo-corydalmine
MAF	muscle aponeurosis fibromatosis
NF-κB	nuclear factor kappa B.
NQO1	NAD(P)H:quinone oxidoreductase1
Nrf2	nuclear factor erythroid 2-related factor 2
OINP	oxaliplatin-induced neuropathic pain
PINP	paclitaxel-induced neuropathic pain
PPARγ	peroxisome proliferator-activated receptor γ
RvD1	resolvin D1
SOD	superoxide dismutase
THCV	tetrahydrocannabivarin
TRP	transient receptor potential
VINP	vincristine-induced neuropathic pain
